# Bone Health for Gynaecologists

**DOI:** 10.3390/medicina61030530

**Published:** 2025-03-18

**Authors:** Angel Yordanov, Mariela Vasileva-Slaveva, Eva Tsoneva, Stoyan Kostov, Vesselina Yanachkova

**Affiliations:** 1 Department of Gynecologic Oncology, Medical University-Pleven, 5800 Pleven, Bulgaria; 2 Research Institute, Medica University Pleven, 5800 Pleven, Bulgaria; sscvasileva@gmail.com (M.V.-S.); drstoqn.kostov@gmail.com (S.K.); v_ess@abv.bg (V.Y.); 3 Department of Breast Surgery, Specialized Hospital for Active Treatment of Obstetrics and Gynaecology “Dr Shterev”, 1330 Sofia, Bulgaria; 4 Department of Reproductive Medicine, Specialized Hospital for Active Treatment of Obstetrics and Gynaecology “Dr Shterev”, 1330 Sofia, Bulgaria; dretsoneva@gmail.com; 5 Department of Gynecology, Hospital “Saint Anna”, Medical University—“Prof. Dr. Paraskev Stoyanov”, 9002 Varna, Bulgaria; 6 Department of Endocrinology, Specialized Hospital for Active Treatment of Obstetrics and Gynaecology “Dr Shterev”, 1330 Sofia, Bulgaria

**Keywords:** bone health, osteoporosis, diagnosis, risk factors, gynaecologist

## Abstract

Osteoporosis, the most common bone disorder, profoundly impacts women’s health, especially during postmenopausal phases. Characterised by diminished bone mineral density (BMD), it increases the risk of fractures, affecting mobility, quality of life, and potentially mortality. The present review analyses the intricate interactions among physiological alterations, diseases, and medications that lead to bone mineral density reduction in women. It underscores the importance of gynaecologists in the prevention, diagnosis, and management of osteoporosis via early risk assessment, suitable hormone treatment, and lifestyle modifications. Essential considerations encompass the categorisation of osteoporosis into primary (age-related) and secondary (attributable to diseases or pharmacological treatments) types, with particular emphasis on predisposing conditions such as premature menopause, hormone deficits, and cancer therapies. The significance of diagnostic instruments such as DXA and novel methodologies like trabecular bone score and quantitative ultrasonography is emphasised for precise evaluation and surveillance. The review also addresses nutritional methods, physical exercise, and pharmaceutical interventions, including hormone replacement therapy (HRT), selective oestrogen receptor modulators (SERMs), and other anti-resorptive drugs, to preserve bone health. This review highlights the important role of gynaecologists in maintaining women’s bone health, promoting a proactive strategy to avert osteoporosis-related complications and enhance long-term results.

## 1. Introduction

Osteoporosis is the most common bone disorder, marked by quantitative and qualitative changes in bone tissue, resulting in reduced density and an increased risk of fractures. The prevalence of this condition cannot be accurately determined. The WHO classification of osteoporosis indicates that the prevalence among postmenopausal Caucasian women in the United States is estimated at 14% for ages 50–59, 22% for ages 60–69, 39% for ages 70–79, and 70% for those aged 80 and older [1].

The World Health Organization (WHO) reports that the condition impacts roughly 6.3% of males over 50 years old and 21.2% of women in the same demographic [2]. This indicates that over 500 million individuals globally could be impacted. A meta-analysis conducted by Salari et al. in 2021 indicates that the prevalence of osteoporosis is 23.1% in women and 11.7% in men [3]. One in three women and one in five men over the age of 50 will experience at least one fracture in their lifetime due to heightened bone fragility.

When addressing the prevalence of osteoporosis, it is crucial to delineate the diagnostic criteria employed for the diagnosis. The World Health Organisation (WHO) defines osteoporosis as a condition diagnosed when bone mineral density (BMD), assessed via dual-energy X-ray absorptiometry (DXA), at the lumbar spine, femoral neck, or whole hip, has a *t*-score of ≤−2.5. A *t*-score between 1.0 and 2.5 standard deviations indicates osteopenia. Pathological fractures may also manifest in people diagnosed with osteopenia, and numerous patients with these fractures lack a BMD test, and cannot be diagnosed with osteoporosis [4,5]. This necessitates the consideration that either the diagnostic criteria for osteoporosis should be broadened or that bone health should be addressed.

Gynaecologists often encounter osteoporosis inadvertently because of the many variables related to its aetiology and the risk factors leading to its onset. This review aims to integrate and summarise available data on women’s bone health for the benefit of specialists in the field.

Diagnosing osteoporosis necessitates a thorough understanding of its related risk factors, along with their proper identification and evaluation. Modifiable and non-modifiable risk factors for osteoporosis are well documented and extensively researched. Non-modifiable risk factors encompass race; gender (primarily female); age; familial history of osteoporosis; a low body mass index (BMI) of 19 or less, maintained at the time of peak bone mass; age of menarche; early menopause; and individuals who have undergone bariatric surgery [6].

Modifiable risk factors encompass a range of eating disorders, including anorexia and bulimia nervosa, extended administration of oral corticosteroids, excessive consumption of coffee, alcohol, and tobacco, cancer patients receiving chemotherapy or hormone therapy, vitamin D3 deficiency, and the use of medications such as proton pump inhibitors, selective serotonin reuptake inhibitors, thiazolidinediones, anticonvulsants, medroxyprogesterone acetate, antiandrogens, calcineurin inhibitors, chemotherapy agents, and anticoagulants [6].

Genetics significantly influence an individual’s skeletal strength, bone microarchitecture, and susceptibility to osteoporosis. Numerous gene variants, or single nucleotide polymorphisms, in a number of genes are linked to a risk of bone loss, indicating that genetic variables have a polygenic effect on bone health. Bone strength and density are influenced by several alleles that contribute to the genetically regulated attainment of peak bone mass in each individual. Examples include the vitamin D3 receptor gene, the oestrogen receptor type A gene, and the collagen type 1 gene, among others The estimated heritable genetic influence on peak BMD is 85%, and a similarly significant genetic predisposition has been identified for bone architectural phenotypes for predicting fracture incidence [7,8]. 

Assessing the risk factors for osteoporosis manifestation and fracture risk is a crucial step in diagnosing patients with the condition and identifying risk groups.

The assessment of fracture risk using BMD alone omits risk factors, prompting the creation of alternative systems that incorporate these variables, like the Garvan fracture risk calculator, QFracture^®^, and FRAX^®^ [9]. The latter is the most prevalent. FRAX^®^ is a computational technique that determines the 10-year likelihood of a major fracture (hip, spine, humerus, wrist) and the 10-year chance of a femoral fracture [10]. This estimate incorporates the following risk factors: age, BMI, prior fractures, parental history of hip fractures, smoking, prolonged use of oral glucocorticoids (irrespective of timing), rheumatoid arthritis, various aetiologies of secondary osteoporosis, and alcohol intake as it pertains to the femoral neck. BMD may be optionally included to enhance fracture risk prediction [11]. The risk of fractures differs globally, hence FRAX^®^ is adjusted based on existing scientific evidence [9]. This algorithm has some serious shortcomings: it does not take into account the number of previous fractures or glucocorticoid doses or daily cigarette and alcohol intake.

## 2. Classification

**Figure 1:**
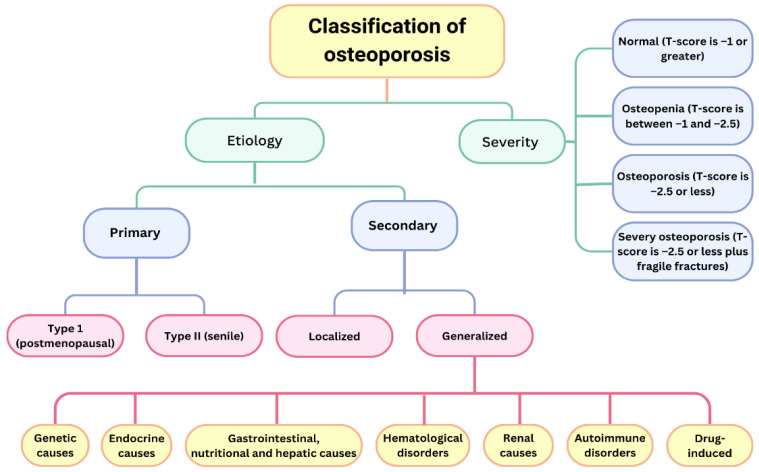
Classification of osteoporosis.

The severity of bone health issues in women is evidenced by the fact that, according to WHO criteria, 30% of postmenopausal Caucasian women in the United States are diagnosed with osteoporosis, while 54% are classified as having osteopenia [7]. Osteoporosis can be classified based on its aetiology and severity [12]. Osteoporosis is classified into primary and secondary types based on its aetiology (Figure 1).

Primary osteoporosis is age-related—BMD decreases with age and is divided as follows [13]:

1. Type I (postmenopausal osteoporosis)—This is more common in women, due to oestrogen deficiency, and mainly affects trabecular bone. Vertebral and distal end radius fractures are more common. This type of osteoporosis is characterized by low calcium levels (due to reduced intestinal absorption and increased renal excretion) and reduced levels of circulating D3 [14].
2. Type II (senile osteoporosis)—This affects both sexes at age >70 years and affects trabecular and cortical bones. Hip and pelvic fractures are more common [14].

Secondary osteoporosis results from a range of conditions, diseases, and medications that diminish bone mineral density and elevate the risk of fractures. These factors influence bone metabolism, either directly or indirectly, and are not associated with ageing [15]. Secondary osteoporosis can be categorised into generalised and localised forms and may occur in both premenopausal and postmenopausal women. About 30% of postmenopausal women exhibit factors contributing to secondary osteoporosis [16,17]. It is essential to identify and address these causes, as they can lead to suboptimal results from conventional osteoporosis treatment [18]. Table 1

It is essential to acknowledge that several causes leading to secondary osteoporosis in women cause a decrease in BMD, hence worsening the condition during menopause.

Comorbidities, such as autoimmune diseases and endocrine disorders, must be assessed for reduced bone mineral density brought on by the disease or its treatment, or for inadequate peak bone mass, if these disorders appeared during puberty.

Further evaluation of patients with diabetes mellitus, hypogonadism, or thyroid dysfunction will facilitate the recognition of the risk associated with alterations in bone mineral density. Several autoimmune illnesses correlate with a marked reduction in bone density and an elevated risk of fractures. In individuals with rheumatoid arthritis, the chance of hip and vertebral fractures escalates two to threefold [19]. In those with systemic lupus erythematosus, the risk of fractures is up by 10–12%, while 25% of those with ankylosing spondylitis have osteoporosis and an increased fracture risk. [19,20]

Drug-induced osteoporosis is a significant contributor to reduced bone mineral density. A number of medications adversely affect bone density, with the impact typically correlating with the duration of exposure and the patient’s age. Table 2 delineates frequently utilised medications that influence bone mineral density.

In gynaecological practice, the long-term use of certain drugs frequently necessitates evaluation due to potential risks to bone health. Consequently, it is essential to undertake additional research on the medication that is utilised in the field of gynaecology clinical practice. Noteworthy findings have been documented regarding the prolonged administration of dienogest in patients with endometriosis. Bone loss predominantly occurs within the initial six months of usage, impacting both the spine and the hip [32]. The correlation between hormonal contraceptive use and bone mineral density is considerable. BMD is comparable or elevated, and fracture rates are analogous or diminished, in women who have used combination oral contraceptives (COCs), relative to non-users [33], something which also applies to mature women. Evidence indicates that the use of combined oral contraceptives (COCs) in teenagers results in diminished bone mineral accumulation, particularly when utilised within the initial three years following menarche [33]. Bone mineral density is reduced at both the spine and hip in users of combined oral contraceptives compared with non-users [34]. Adolescents who discontinue the use of combined oral contraceptives exhibit a lesser increase in spinal bone mineral density compared with non-users at 12 and at 24 months post-discontinuation [35]. These effects are absent with transdermal and progestin-only contraceptives. The sole exception is DMPA, wherein BMD loss is more pronounced in teens compared with adult women. The reductions in teenagers occur most swiftly over the initial 1–2 years of usage [34]. Upon cessation of DMPA, BMD significantly or totally recovers [36]. The fracture risk linked to hormonal contraception use throughout adolescence remains ambiguous due to insufficient evidence on fracture occurrence. Combined oral contraceptives containing 30 mcg of ethinyl oestradiol, as opposed to lower-dose formulations, are believed to confer a protective effect on bone health [34].

All of these diseases, conditions, and the use of various medications may result in significant bone mineral density losses when a woman goes through menopause. As a result, the gynaecologist needs to provide more tailored therapy for these women, both during the transition into menopause and during menopause itself. In terms of the administration of hormone therapy to young women, this requires special attention and caution.

Several factors related to secondary osteoporosis warrant consideration.

1. Fracture risk cannot be assessed solely based on BMD values.
2. Fractures may also occur in patients with a *t*-score of ≤−2.5.
3. Optimise the treatment of the underlying condition.
4. Medications that result in a reduction of BMD should be administered at minimal therapeutic doses.
5. Address nutritional deficiencies.
6. Address risk factors: smoking, alcohol consumption, caffeine intake, low body mass index (BMI), and decreased dairy product consumption.

## 3. Bone Loss Periods in a Woman’s Life

The bone density of a woman is predominantly established during the stages of puberty and shortly thereafter. Peak bone mass is typically attained by approximately age 25, after which a progressive decline in bone density commences around age 30.

Physiological phases in a woman’s life, such as pregnancy and lactation, are linked to alterations in BMD [37]. The reduction in BMD is temporary and is later recovered. No evidence exists to suggest that there is an altered fracture risk during menopause; however, this possibility should not be dismissed, particularly in multiparous women and those who have breastfed for extended durations, especially when other predisposing factors such as diseases, corticosteroid therapy, or early menopause are present.

As a woman enters menopause, bone loss increases rapidly and it should be noted that this loss begins between the ages of 30 and 40 [38]. These alterations may result in osteoporosis 10 to 15 years post-menopause [39]. The maintenance of BMD is an ongoing process involving absorption and resorption, resulting in the remodelling of 10% of bone each year. As age advances, this equilibrium tilts towards heightened bone resorption. Approximately 10% of BMD is diminished after menopause, with an estimated 50% of women experiencing a loss of up to 20% BMD. Approximately 25% of postmenopausal women are categorized as rapid bone losses, experiencing an annual loss above 3.5% [40]. This culminates in an elevated risk of fractures, which renders osteoporosis a disease of considerable socioeconomic significance.

Osteoporotic fractures result in diminished mobility and independence, decreased quality of life, and the onset of severe consequences such as pneumonia or thromboembolic illness, which may be deadly; 20–30% of patients with a hip fracture die within the first year [41]. Consequently, prone detection and treatment of these patients is essential. Fractures typically occur in a specific order based on bone type: wrist, rib, and humeral fractures are common around menopause; vertebral fractures generally arise 10–15 years later, and hip fractures typically occur around the age of 70.

**Figure 2:**
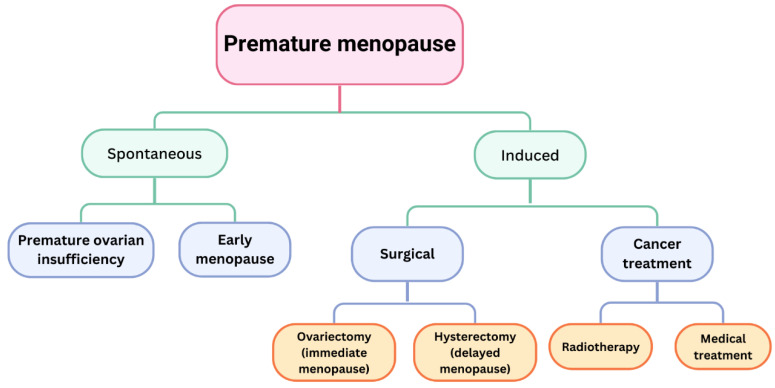
Causes of premature menopause.

An essential inquiry pertains to the implications for patients experiencing premature menopause and if the aetiology—spontaneous or iatrogenic—correlates with BMD (Figure 2).

Menopause that occurs before the age of 40 is termed premature ovarian insufficiency (POI), impacting 1–3% of women, whereas menopause occurring between the ages of 40 and 44 is referred to as early menopause (EM), affecting 10% of women [42]. POI is predominantly idiopathic, but may also result from autoimmune disorders, hereditary factors, infections, inflammatory conditions, enzyme shortages, or metabolic abnormalities. Both diseases result in diminished BMD, an elevated risk of osteoporosis, and consequently, a higher risk of fractures [43]. Early menopause (EM) nearly doubles the risk of osteoporosis compared with standard menopause [44]. Certain investigations do not identify an elevated fracture risk in individuals with POI, attributing this observation to the more prevalent utilisation of HRT among these patients [45]. A separate study indicated that a one-year reduction in the age of the final menstrual period results in a 5% elevation in fracture risk [46].

The question of whether induced premature menopause affects bone mineral density differently is intriguing. The hormonal changes in these patients differ in intensity from those observed in patients experiencing natural menopause [47]. Induced menopause occurs as a result of bilateral oophorectomy in premenopausal women or as a consequence of treatments for malignant disorders, including chemotherapy and radiotherapy. Alterations in ovarian function due to chemotherapy and/or radiotherapy may be reversible or permanent, depending upon the patient’s age, treatment dosage, and duration. Surgical menopause transpires promptly following bilateral oophorectomy; nevertheless, evidence indicates that the removal of the uterus, fallopian tubes, or both will result in menopause within five years, irrespective of the retention of one or both ovaries [48].

Menopause occurs more quickly if only one ovary is preserved [49]. The literature lacks data on the impact of various surgical operations on BMD. Bilateral oophorectomy in women under 45 years is a risk factor for osteoporosis, and such surgery after natural menopause elevates fracture risk compared with women with intact ovaries. Matsuno et al. documented a reduction of more than 6% in bone mineral density over the initial year following bilateral oophorectomy [50]. These findings have been substantiated in other investigations, including those involving patients who underwent bilateral oophorectomy as a component of the surgical management of gynaecological neoplasms [50] or in individuals with breast cancer [51].

BMD loss in patients receiving cancer treatment is significantly more dramatic and faster, potentially reaching levels 10 times greater than the normative values for their age [52]. The likelihood of developing osteoporosis is elevated in breast cancer patients undergoing treatment with aromatase inhibitors, as well as in perimenopausal and postmenopausal women [52].

Bilateral oophorectomy and certain cancer treatments are thought to induce early menopause, resulting in heightened bone mineral density loss, which may subsequently lead to osteoporosis and an elevated risk of fractures. These occurrences also adversely affect women who have already undergone menopause. The premature start of menopause is critically significant for bone health, so it is pertinent to refer to it as premature menopause. The aetiology is significant, and based on this, gynaecologists should be equipped to address these disorders.

## 4. Diagnostic and Screening Methods

The gold standard for assessing BMD is dual-energy X-ray absorptiometry (DEXA) of the vertebral bodies and proximal femur. Peripheral dual-energy X-ray absorptiometry (pDXA) of the forearm is employed as a supplementary technique. Quantitative ultrasonography (QUS) of cancellous bone or other sites (radius, tibia) is utilized solely as a screening tool to assess the risk of osteoporosis and fractures. The diagnosis of osteoporosis is determined by evaluating the *t* and Z scores derived from the DEXA test, together with the analysis of bone turnover markers. The Z-score is utilized to distinguish the condition in young individuals, specifically women under 50 years of age, and in instances of probable secondary osteoporosis.

- Normal bone density: *t*-score > −1.0 SD.
- Osteopenia: *t*-score ≥ −1.0 SD < −2.5 SD.
- Osteoporosis: *t*-score ≤ −2.5 SD.
- Severe osteoporosis: *t*-score ≤ −2.5 SD and previous osteoporotic fracture

DXA has some disadvantages—it measures only density, not bulk density (in milligrams per cubic centimetre), BMD is dependent on bone size and may overestimate fracture risk in individuals with small body frames, and degenerative diseases of the spine and hip may give a lower fracture risk than the actual one [53].

DXA is not consistently a reliable tool, as additional bone properties, such as microarchitecture, bone remodelling, and bone geometry, all influence fracture risk [54]. This required the establishment of a new measure in 2010—the trabecular bone score (TBS) [55]. The findings are elucidated as follows: TBS > 1.310 indicates normal bone microarchitecture and a low fracture risk; TBS values between 1.23 to 1.31 signify moderately impaired microarchitecture; and TBS < 1.230 reflects degraded microarchitecture and an elevated fracture risk [56]. TBS is advised for evaluating the risk of osteoporotic fractures in postmenopausal women [57].

BMD assessment can be conducted through computed tomography (CT) or magnetic resonance imaging (MRI). Both methods, however, require greater energy expenditure from the patient, demand more time, and incur higher costs. Consequently, these methods are not employed in standard practice; however, substantial evidence indicates that CT scans conducted for other purposes, which also assess the spine, can serve as a means for opportunistic screening for osteoporosis [58,59]. Quantitative computed tomography (QCT) is now being implemented in clinical practice for the measurement of BMD in both central and peripheral bone systems [60].

Ultrasound systems designed for assessing bone health have been in development for over 40 years, with the objective of addressing certain limitations of DXA [61]. Ultrasound offers a qualitative assessment of bone rather than a quantitative one; it measures the speed of sound (SOS), which can indirectly evaluate bone porosity, a known risk factor for fractures. SOS measurements indicate various bone properties, including density, elasticity, cortical thickness, and, to a lesser degree, microarchitecture. This offers a more comprehensive understanding of bone fragility compared with bone mineral density assessed by DXA alone [62].

Research demonstrates that the sensitivity and specificity of calcaneus quantitative ultrasound (QUS) are insufficient to replace dual-energy X-ray absorptiometry (DXA) for osteoporosis screening in specific patient populations. Nonetheless, it may serve as a useful preliminary screening tool by which to identify patients with a high or low likelihood of osteoporosis before undergoing DXA. Thus, calcaneus QUS reduces the need for referral to DXA [63]. This method’s primary limitation is its analysis of a single site, which may lead to false negative results; however, its simplicity is a significant advantage. Some studies indicate that this approach is effective for identifying osteoporosis in the spine and femur [64].

**Figure 3:**
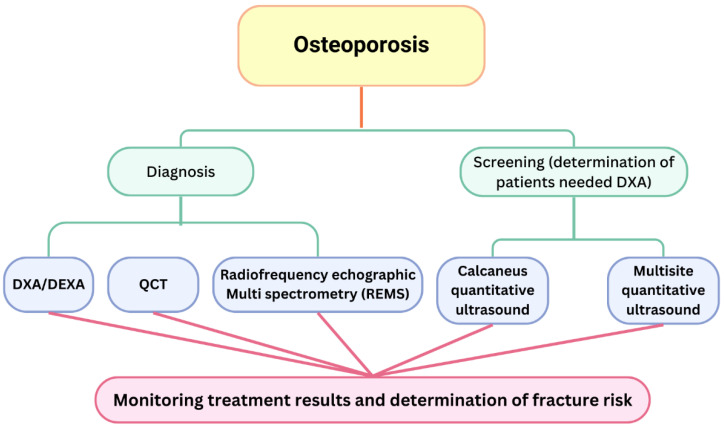
The utilization of various diagnostic methods in the management of bone health.

Several multisite ultrasounds have been created to evaluate bone strength and fracture risk; however, direct comparison with DXA is unfeasible due to the measurement of different parameters. Additionally, there is a scarcity of studies comparing calcaneal ultrasound with multisite quantitative ultrasound. Several studies have indicated that tibial measurements may serve as a diagnostic tool for osteoporosis, while others suggest that both tibial and radius measurements can predict the risk of hip, vertebral, and overall fractures in postmenopausal women, considering various risk factors. QUS is not advised for initiating or monitoring treatment; however, it may serve as a triage tool to identify patients who require DXA testing. A novel technique, radiofrequency echographic multi spectrometry (REMS), has been developed in recent years. For this method, the proximal femur and lumbar spine are both scanned. This method is applicable for diagnosing osteoporosis and assessing fracture risk [65]. Each of these methods possesses distinct value, significance, and applicability in evaluating bone health in women, as QUS can assess fracture risk at specific locations (Figure 3).

## 5. Prevention of Osteoporosis

In terms of osteoporosis prevention and treatment, a proper nutritional and physical activity routine should always be the first step.

Nutrition is of utmost importance for bone health. Proper calcium and vitamin D supplementation is essential. Supplementation is significantly influenced by the patient’s age and concomitant conditions. Generally, with advancing age, the absorption of numerous micro- and macronutrients is insufficient due to alterations in the gastric and intestinal mucosa. This also pertains to gastrointestinal tract illnesses. Additionally, with advancing age, the synthesis of vitamin D3 markedly diminishes due to the ageing of the skin. Nonetheless, air pollution, which obstructs the penetration of essential sunshine into the skin, along with certain dermatological conditions, may also correlate with vitamin D3 deficiency.

The optimal daily calcium intake is 1000 mg for premenopausal women and 1200 mg for postmenopausal women [66]. Calcium-rich foods encompass milk, cheese, and other dairy products; green leafy vegetables such as broccoli, cabbage, and okra, excluding spinach; soybeans; tofu; plant-based beverages (e.g., soy milk) fortified with calcium; almonds; bread and products produced with fortified flour; and fish. Calcium supplementation is primarily contingent upon the patient’s general health status. The daily requirement for vitamin D is between 800 IU and 2000 UI according to the individual patient characteristics [66,67]. Foods rich in vitamin D include oily fish, such as salmon, sardines and mackerel; egg yolks; fortified foods, such as some fat spreads; and breakfast cereals [68].

Calcium supplementation alone does not diminish fracture risk [69]; however, the conjunction of calcium and vitamin D decreases by 5 to 15% for all fractures and 13 to 30% for hip fractures [70]. Increased magnesium consumption enhances bone mineral density at the hip and femoral neck, although it does not alter fracture risk [71,72]. Increased protein consumption enhances bone mineral density and diminishes fracture risk [73]. The intake of sugar-sweetened beverages, particularly carbonated drinks, correlates with diminished BMD [74], but the use of tea, especially green tea—likely attributable to flavonoids and polyphenols—exhibits a contrasting effect [73]. The consumption of dairy products, particularly fermented varieties, diminishes the incidence of hip fractures, but a decreased intake of fruits and vegetables elevates this risk [73].

In recent years, there has been increasing evidence that probiotics play a major role in bone health and may affect postmenopausal BMD loss. In animal models, probiotic treatment has been shown to significantly improve BMD in ovariectomized animals and to show a trend toward increased bone formation and decreased bone resorption [75]. Another study has reported that probiotics can increase BMD in postmenopausal women [76], and a meta-analysis concluded that this process is more pronounced in women with osteopenia than osteoporosis [77].

Physical activity is of great importance. Mechanoreceptors in bone are stimulated by external forces, hence facilitating bone remodelling. This suggests that particular forms of exercise may influence bone mineral density at specific locations in the skeleton of menopausal women. The research presents conflicting evidence: a 2017 meta-analysis indicated that the combination of at least two distinct forms of exercise preserves BMD in postmenopausal women at various locales [78]. Bloch-Ibenfeldt has reported that 1 year of heavy resistance training improves short-term bone formation in healthy elders, though this is not confirmed at long-term follow ups [79]; however, other meta-analyses do not corroborate this conclusion [80,81]. All reports indicate that physical activity enhances patients’ general fitness, elevates quality of life, and diminishes fracture risk by lowering the likelihood of falls and by augmenting muscle strength.

Osteoporotic fractures in women occur in a defined chronological order: wrist, rib, and humerus fractures manifest around the time of menopause, vertebral fractures arise 10–15 years subsequently, and hip fractures occur at a somewhat older age. This sequence is contingent upon peak bone mass achieved at the conclusion of puberty and the escalation of bone remodelling at the onset of menopause [82]. Until the onset of the century, HRT was extensively utilised globally for osteoporosis prevention and it is well known that HRT significantly improves the quality of life for menopausal women [83]; however, following the release of the Women’s Health Initiative (WHI) findings, which indicate heightened risks of cardiovascular and cerebrovascular diseases, along with breast cancer, the utilisation of HRT diminished, resulting in a rise in fracture incidence over the past decade [84].

HRT results in a fast decline in bone resorption within 3 to 6 months of initiating treatment, followed by a stabilization of bone turnover approximately 6 to 12 months post-therapy initiation, persisting as long as oestrogen therapy is sustained. BMD mostly rises during the initial year of treatment, experiences a lesser increase in the subsequent year, and then stabilizes as long as therapy persists. This effect is dependent on the dosage of oestrogen and is more pronounced with low-dose oestrogens compared with regular daily doses (i.e., 0.625 mg conjugated equine oestrogens, 2 mg 17β-estradiol, or 50 mg transdermal 17β-estradiol). Currently, the use of low-dose oestrogens is advised, as they have demonstrated efficacy in alleviating vasomotor symptoms, preventing vaginal atrophy, and mitigating early postmenopausal bone loss [85,86,87,88,89,90].

The influence of oestrogen on bone is independent of its method of delivery (oral or transdermal), the inclusion of progestogens, or the baseline level of bone mineral density and age at the commencement of treatment. HRT diminishes the risk of vertebral fractures by approximately 40%, hip fractures by 30%, and the risk of all osteoporotic fractures by 20–30% in comparison with calcium and vitamin D treatment [82]. This effect is more significant prior to the age of 60 [91] and remains unaffected by variables such as age, body mass index, smoking status, personal or familial fracture history, total calcium consumption, and baseline BMD. The protective benefit may wane five years post-treatment cessation [92,93]; however, bone mineral density in women who underwent prolonged hormone replacement therapy remains elevated after discontinuation, relative to those who received a placebo [94]. While HRT is regarded as the most efficacious and evidence-supported treatment for women at minimal fracture risk [95], there is no justification for administering HRT to asymptomatic menopausal women with osteopenia [96]. The U.S. Preventive Services Task Force (USPSTF) does not advocate using hormone replacement therapy (HRT) exclusively for the prevention of osteoporosis [97]. It is suitable for women experiencing vasomotor symptoms who are either under 60 years of age or within 10 years of their last menstrual period [98].

An exception can be made for patients with primary ovarian insufficiency or endometriosis [98]. BMD declines significantly after discontinuation of HRT (1.5–2% year) [99]; however, it stays above pre-treatment levels [94]. The risk of fracture does not elevate in the short term following the cessation of hormone replacement therapy (HRT) [100].

**Figure 4:**
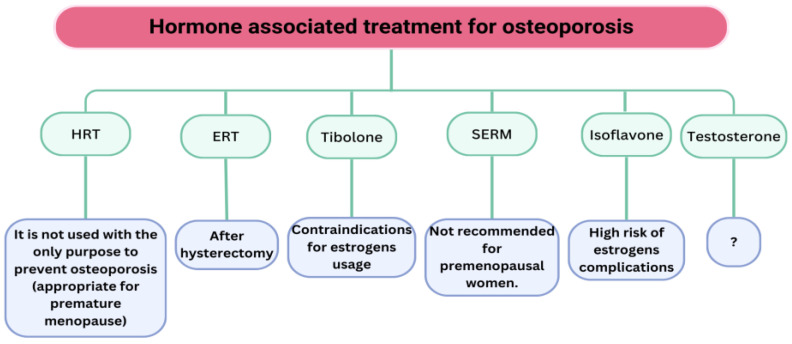
Utilization of hormone-based therapy for osteoporosis.

Women who have had hysterectomies are prescribed oestrogen-only therapy (ERT). In light of the numerous negative effects associated with HRT, other alternatives are being explored. An instance is the application of a hormonal intrauterine device containing progestogen alongside transdermal oestrogen [101]. Additional possibilities are SERMs, Tibolone, and phyto-oestrogens (Figure 4). SERMs exert a similar mechanism of action on bone as oestrogens by promoting osteoclast apoptosis, although they do not produce the same effects on the endometrium and breast.

The FDA has approved two medications: raloxifene, utilised for the treatment and prevention of osteoporosis, and bazedoxifene, which is indicated solely for the prevention of osteoporosis when administered in conjunction with oestrogen. These medications diminish the likelihood of vertebral fractures, but not hip or nonvertebral fractures [102]. Discontinuing SERMs results in fast bone deterioration and a heightened likelihood of fractures [103]. These medications are advised for relatively young women who exhibit a high risk of vertebral fractures and a low risk of nonvertebral fractures and deep vein thrombosis [104]. Similar to oestrogen therapy, prolonged usage of SERMs is discouraged, necessitating an alternative medication upon SERM cessation [104].

Tibolone serves as an alternate treatment for osteoporosis in postmenopausal women. It is equally effective as hormone replacement therapy, although it entails less adverse effects. It enhances bone mineral density in a way akin to oestrogen therapy and is linked to fewer side effects compared with oestrogen therapy [105]. A daily intake of 106 mg (range, 40–300 mg) of isoflavones for 6–24 months demonstrates a minor yet statistically significant beneficial impact on BMD at the lumbar spine, femoral neck, and hip, indicating that soy isoflavones are helpful in mitigating bone loss post-menopause.

Testosterone therapy presents a compelling option for the prevention of osteoporosis and fracture risk in postmenopausal women. Testosterone levels diminish during menopause; nevertheless, conclusive data regarding the impact of testosterone therapy on bone mineral density in postmenopausal women is lacking [106]. Elraiyah et al. have reported that testosterone does not affect BMD in any location [107]. Miller et al. [108] observed a notable enhancement in hip BMD in patients receiving testosterone therapy compared with those undergoing hormone replacement therapy (HRT) alone. Conversely, Barrett-Connor et al. [109] documented an increase in BMD at both the hip and lumbar spine with testosterone treatment relative to placebo.

Davis et al. [110] compared estradiol therapy to estradiol combined with testosterone implants, finding a substantial enhancement in BMD for the entire body, L1–L4 vertebrae, and trochanter in the latter cohort. A further study indicated that the administration of a testosterone pellet, either singularly or in conjunction with low-dose estradiol (E2), led to enhanced BMD in the spine and hip, with more significant improvements shown when both were used together [111].

**Figure 5:**
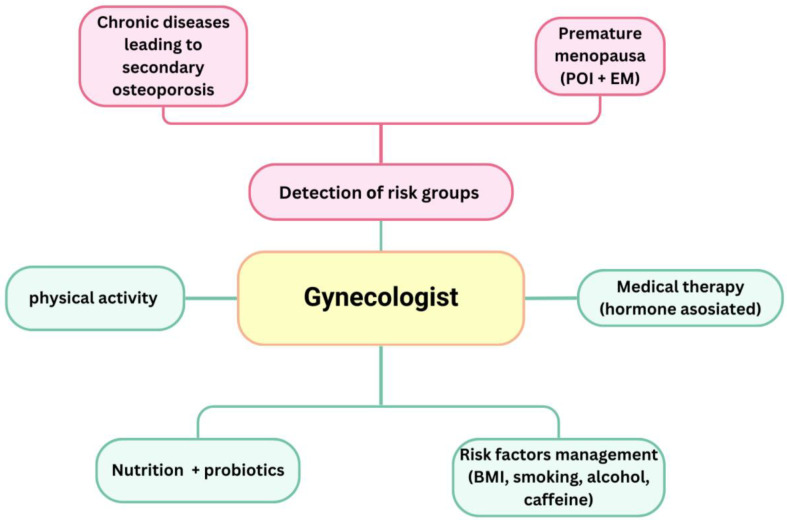
The role of the gynaecologist in osteoporosis prevention.

Contemporary medicine and diagnostic methodologies facilitate the early identification, treatment, and prevention of various socially significant disorders, including osteoporosis. The silent progression of osteoporosis establishes circumstances that lead to a lasting decline in patients’ quality of life, enduring impairment, and potentially death. Factors such as lifestyle, environmental influences, stress, general radiation exposure, genetic predispositions, and numerous disorders contribute to premature ovarian failure, early onset of menopause, and the occurrence of menopause in progressively younger women. Significant declines in hormone levels result in substantial alterations in bone mineral density. Gynaecologists are typically the first healthcare providers to assess these individuals, mostly due to issues associated with menopausal alterations. Consequently, these professionals serve as a foundational resource for the early identification of patients exhibiting risk factors for osteoporosis. It is imperative for gynaecology professionals to identify and evaluate risk factors and to be acquainted with diagnostic algorithms and methodologies, as well as contemporary preventative and treatment alternatives (Figure 5).

## 6. Conclusions

Multiple factors in a woman’s life can influence BMD. Complications can occur despite adequate bone mineral density; thus, it is essential to address whole bone health rather than exclusively concentrating on osteoporosis and osteopenia. Gynaecologists are essential in protecting women’s bone health, bearing the primary duty for its prevention. To perform this role effectively, clinicians must acquire specialised knowledge, as follows:

- To understand the physiological factors contributing to a reduction in BMD and to identify when these women are at risk for developing osteoporosis.
- To identify risk groups affected by disorders that result in secondary osteoporosis and administer appropriate treatment.
- To assess the risk to bone health in young women receiving hormone therapy for gynecological conditions, particularly in adolescents utilizing oral contraceptives within three years post-menarche.
- To effectively manage women with POI and EM, HRT should be administered to women with POI until the age of natural menopause, contingent upon the underlying aetiology.
- To effectively manage women after gynecological procedures, oophorectomy results in accelerated bone mineral density loss, while hysterectomy induces menopause within four years.
- To effectively monitor patients with oncological conditions, particularly breast cancer.
- To understand the capabilities of various imaging modalities employed for diagnosing bone density alterations and their applicability in screening and therapy monitoring (calcaneus and multisite QUS).
- The application of HRT and SERM for preserving bone health in relatively younger women should be limited in duration, and HRT should not be administered to asymptomatic women (lacking climacteric symptoms) as a treatment for osteoporosis.

## Figures and Tables

**Table 1:** Secondary osteoporosis causes.

Genetic diseases	Idiopathic juvenile osteoporosis Osteogenesis imperfecta Osteoporosis—pseudoglioma syndrome (OPPG) Hereditary enzyme deficiencies Turner syndrome
Endocrine diseases	Hyperthyroidism Hypothyroidism Diabetes mellitus Hypogonadism Hyperparathyroidism Cushing syndrome Growth hormone deficiency Acromegalia
Gastrointestinal diseases	Celiac disease Inflammatory bowel disease Hemochromatosis Chronic liver diseases Malabsorption syndrome
Bariatric surgery	Gastric bypass surgery
Eating disorders	Anorexia neurosa Bullimia neurosa
Haematological disorders	Monoclonal gammopathy of uncertain significance (MGUS) multiple myeloma Systemic mastocytosis Beta thalassemia major
Renal diseases	Idiopathic hypercalciuria Renal tubular acidosis. Chronic kidney disease (CKD)
Autoimmune diseases	Rheumatoid arthritis Systemic lupus erythematosus Ankylosing spondylitis Multiple sclerosis
Vitamin D3	Vitamin D3 deficiency
Infection diseases	Tuberculosis

**Table 2:** Frequently used medications associated with reduced bone mineral density (BMD).

Glucocorticoids	Up to 3 months use of high doses (the process is reversible)
Thyroid hormones	Thyrotoxicosis facticia
Aromatase inhibitors	Prolonged use results in diminished oestrogen levels, thereby causing bone loss. [21,22,23]
Medroxyprogesterone acetate	Inhibits gonadotropin secretion, suppresses ovarian oestrogen production, and leads to a decrease in BMD (process is reversible) [24]
Gonadotropin releasing hormone (GnRH) agonists	Suppress oestrogen levels and thus lead to a decrease in BMD; significant BMD loss can occur after 3–6 months of therapy [25,26,27] (the process is reversible)
Antidepressants	More pronounced effect in patients over 50 years of age: they reduce BMD and increase fracture risk [28]
Anticonvulsants	Greater decrease in BMD in women aged ≥65 years
Thiazolidinediones	Associated with an increased risk of fractures
Drugs with actions on the immune system	Calcineurin inhibitors—unknown mechanism Antiretroviral therapy—3- to 7-fold increase in the risk of osteoporosis and increased risk of fractures [29]
Anticoagulants	One third of patients taking long-term heparin have reduced BMD [30]
Diuretics	Loop diuretics lead to reduced BMD and an increased risk of osteoporotic fractures [31].

## Data Availability

The authors declare that all related data are available from the corresponding author upon reasonable request.
